# Speed and accuracy of saccades in patients with glaucoma evaluated using an eye tracking perimeter

**DOI:** 10.1186/s12886-020-01528-4

**Published:** 2020-06-30

**Authors:** Andrew J. Tatham, Ian C. Murray, Alice D. McTrusty, Lorraine A. Cameron, Antonios Perperidis, Harry M. Brash, Brian W. Fleck, Robert A. Minns

**Affiliations:** 1 University of Edinburgh, Edinburgh, UK; 2 Princess Alexandra Eye Pavilion, 45 Chalmers Street, Edinburgh, EH3 9HA UK; 3 Glasgow Caledonian University, Glasgow, UK; 4 Royal Hospital for Sick Children, Edinburgh, UK

**Keywords:** Glaucoma, Visual field, Perimetry, Eye tracking

## Abstract

**Background:**

To examine the speed and accuracy of saccadic eye movements during a novel eye tracking threshold visual field assessment and determine whether eye movement parameters may improve ability to detect glaucoma.

**Methods:**

A prospective study including both eyes of 31 patients with glaucoma and 23 controls. Standard automated perimetry (SAP) and eye tracking perimetry (saccadic vector optokinetic perimetry, SVOP) was performed. SVOP provided data on threshold sensitivity, saccade latency, and two measures of accuracy of saccades (direction bias and amplitude bias). The relationship between eye movement parameters and severity of glaucoma was examined and Receiver Operating Characteristic curves were used to assess ability to detect glaucoma.

**Results:**

Patients with glaucoma had significantly slower saccades (602.9 ± 50.0 ms versus 578.3 ± 44.6 ms for controls, *P* = 0.009) and reduced saccade accuracy (direction bias = 7.4 ± 1.8 versus 6.5 ± 1.5 degrees, *P* = 0.006). There was a significant slowing of saccades and saccades became less accurate with worsening SAP sensitivity. Slower saccades were associated with increased odds of glaucoma; however, the AUC for saccade latency was only 0.635 compared to 0.914 for SVOP sensitivity.

**Conclusion:**

Patients with glaucoma had significant differences in eye movements compared to healthy subjects, with a relationship between slower and less accurate eye movements and worse glaucoma severity. However, in a multivariable model, eye movement parameters were not of additional benefit in differentiating eyes with glaucoma from healthy controls.

## Background

Glaucoma is a leading cause of global blindness, with an estimated prevalence of 3.54% among those aged 40 to 80 years [1]. In 2013, 64.3 million people were estimated to be affected worldwide and this number is projected to increase to 111.8 million by 2040 [1]. Assessment of the visual field is essential for the detection and monitoring of glaucoma, with standard automated perimetry (SAP) the gold standard. SAP is a form of static threshold perimetry which uses a white stimulus on a white background to determine differential light sensitivity. Though the duration of SAP testing can be reduced by modifying testing strategies, patients often find perimetry difficult to perform [2]. In addition, several studies have shown only weak correlation between SAP and the ability to perform vision-related tasks of daily living, suggesting that SAP fails to fully capture the impact of glaucoma on visual function and quality of life [3] [4]. Improved methods of assessment of visual function are needed.

Glaucomatous visual field loss has been shown to affect eye movement patterns during tasks such as reading [5] [6], driving [7], watching television [8], viewing scenes [9, 10], recognizing faces [11], and performing visual search [12]. In addition, there is evidence that eye movements may be altered even before visual field loss is detected [13] [14]. However, there remains uncertainty concerning how exactly eye movements are affected in glaucoma. Whereas some studies have reported patients with glaucoma to have altered saccade rate and amplitudes [7], this has not been a universal finding [12], perhaps due to differences in task, disease severity, and possibly compensatory strategies.

We have recently described saccadic vector optokinetic perimetry (SVOP), a new method of automated perimetry which determines threshold visual field sensitivity using eye tracking [15–18]. SVOP uses an eye tracker to assess gaze responses to stimuli presented on a display screen. An algorithm determines automatically if the stimuli have been seen based on the direction and amplitude of the gaze response. There is no requirement for the patient to use a chin rest or to press a button to register a response. Previous studies have shown strong agreement between threshold sensitivity values obtained with SAP and SVOP [17, 18], and have reported patients to prefer the SVOP experience [17, 18]. In addition to providing information on threshold sensitivities, unlike SAP, SVOP captures data on patterns of eye movement, which may provide additional information relevant to glaucoma. The purpose of this study was to examine the latency and accuracy of saccadic eye movements recorded by SVOP during threshold visual field assessment and determine whether this information may improve ability to detect glaucoma.

## Methods

This was a prospective study including both eyes of 54 subjects, including 31 patients with glaucoma and 23 healthy participants. Participants with glaucoma were recruited from the glaucoma clinic at the Princess Alexandra Eye Pavilion, Edinburgh, Scotland. Healthy participants were recruited through the Scottish Health Research Register (SHARE), a register of volunteers interested in research [15]. Participants provided written informed consent and study methods were approved by the South-East Scotland Research Ethics Committee (reference 13/SS/0045). The study adhered to the tenets of the Declaration of Helsinki.

Patients attending the glaucoma clinic underwent a comprehensive ophthalmic examination, including best-corrected visual acuity, slit lamp biomicroscopy, intraocular pressure (IOP) measurement using Goldmann applanation tonometry, gonioscopy and dilated fundoscopy. The diagnosis of glaucoma was made by a glaucoma specialist, based on the presence of glaucomatous changes to the optic or retinal nerve fibre layer and a glaucomatous visual field defect on SAP using the Humphrey visual Field Analyzer (HFA 750i) SITA Fast 24–2 test (Carl Zeiss Meditec, Inc., Dublin, CA) [16, 17]. Patients with non-glaucomatous conditions that might affect the visual field were excluded. Healthy participants were required to have no history of significant eye disease, no known history of visual field defect and no known ocular or systemic conditions that might affect the visual field.

All participants were tested using SAP using the Humphrey Field Analyser (HFA, 750i, *Carl Zeiss Meditec, Dublin, CA*). The 24–2 test pattern and SITA Fast algorithm were selected. SVOP was performed at the same visit using a SVOP research device, described in detail previously [16, 17]. All patients completed SAP and SVOP in both eyes, with testing order randomized. SAP tests with ≥15% false positives or ≥ 20% fixation losses were considered unreliable and excluded. SVOP does not provide information about false positives or fixation losses as a stimulus is only shown when the patient is fixating on the previous stimulus.

### Saccadic vector Optokinetic Perimetry (SVOP)

The threshold SVOP device consists of a personal computer with a 24″ high-resolution Liquid Crystal Display (LCD) screen (Eizo ColorEdge CG243W, Hakusan, Japan) and an eye tracker (X2–60, Tobii Technology, Stockholm, Sweden) [16, 17]. The screen is pre-calibrated using a look-up table pairing the grey-levels of each pixel to the corresponding required background (10Cd/m^2^) and stimulus luminance levels [18]. The eye tracker measures changes in eye movements related to stimuli presented on the display screen. A computerised algorithm was devised to determine if the stimuli had been seen based on the direction and amplitude of the patient’s gaze response. The eye tracker also provided ‘real-time’ data on eye location meaning that the size and position of the stimuli could be automatically adjusted to compensate for changes in the patient’s position from the screen during testing. This meant that patients did not need to place their chin on a rest and were free to move their head during testing. As responses to stimuli were detected automatically from eye movements, there was no need for the patient to press a response button [16, 17].

**Fig. 1:**
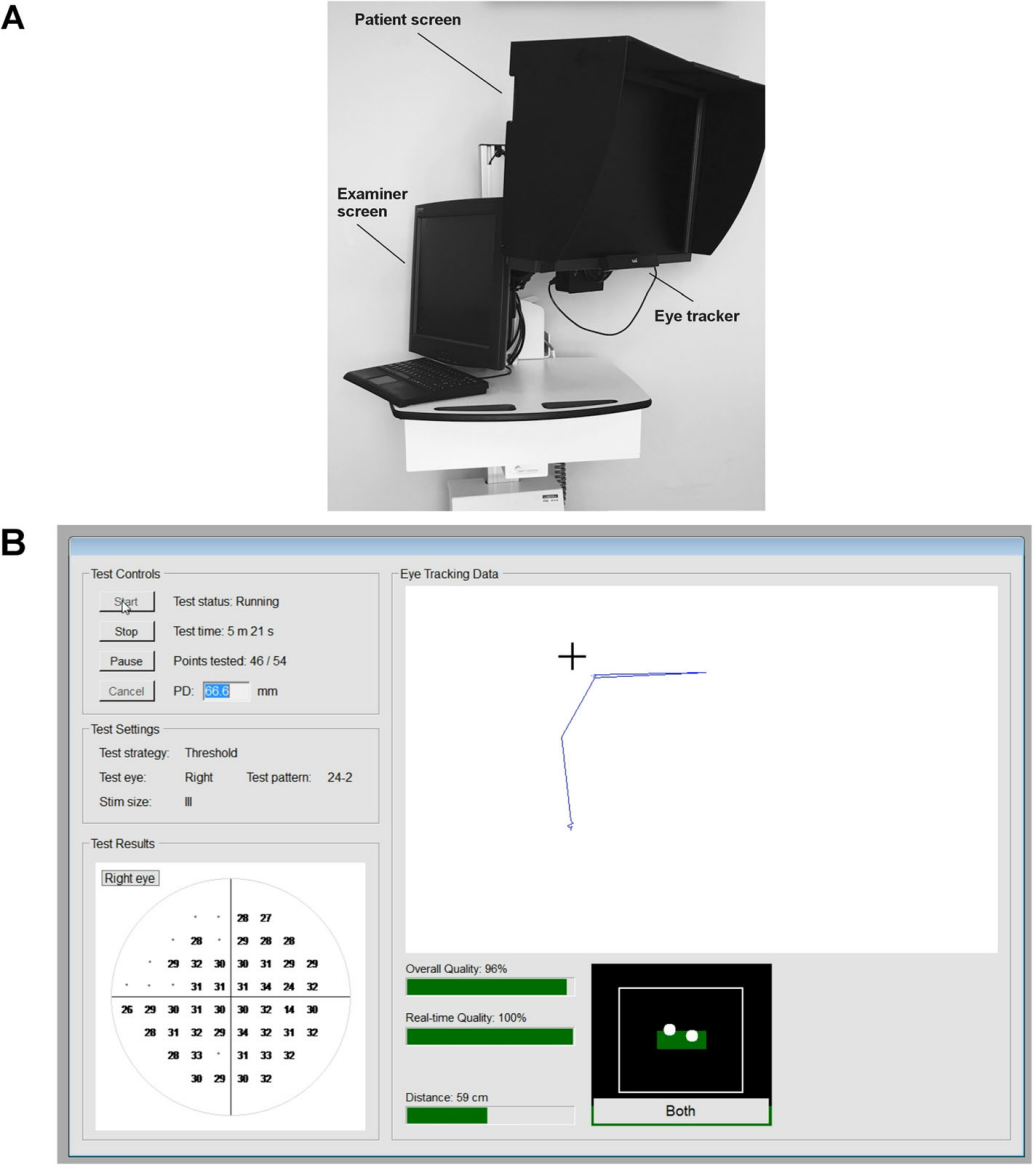
(**a**) Photograph of the saccadic vector optokinetic perimetry (SVOP) device showing the examiner screen, patient screen and eye tracker. (**b**) Screenshot of the SVOP examiner screen during testing showing live eye tracking data and test results

Participants were seated in front of the LCD screen with their eyes aligned with the screen’s centre, initially positioned 55 cm from the screen (Fig. 1A). Each eye was tested separately, with custom made test spectacles used to occlude the non-test eye using darkened infrared bandpass filter. The filter allowed the eye tracker to detect the position of both eyes. Each patient was provided with a 20 s demonstration of the SVOP test, which was followed eye-tracker calibration. During testing the patient was instructed to follow their natural reaction to fixate towards any peripheral stimulus perceived, while the technician monitored a second screen showing a live feed of the eye tracking (Fig. 1B) [16, 17]. Whether or not the stimulus had been seen was determined based on the direction and amplitude of the change in eye position relative to fixation spot and stimulus. Changes to position of gaze were monitored at a 50 Hz sample rate. The start of a fixation change was defined as the start point of a > 50 pixels gaze change and the end location of a fixation change was defined by the point at which 5 consecutive gaze data samples were separated by a distanced of < 50 pixels, occurring after the detection of a fixation change start point. Stimuli were equivalent to Goldmann size III and each stimulus was presented for 200 ms using coordinates equivalent to the SAP 24–2 test pattern [15, 16].

The screen was calibrated using a look-up table pairing the grey-levels of each pixel to the corresponding required background (10Cd/m^2^) and stimulus luminance levels [15, 16]. Stimuli luminance levels replicated the luminance values corresponding to the 14 to 40 dB range tested with SAP. Luminance values greater than 14 dB were not tested as the LCD display was not able to accurately produce values above this. A 4–2 bracketing strategy was used to assess thresholds, beginning with testing at four ‘seed’ locations (one in each quadrant). These were then used to determine the stimulus luminance levels for starting quantification of threshold values at neighbouring visual field test point locations [15, 16]. Metrics obtained from SVOP included threshold sensitivity, two measures of saccade accuracy (direction bias and amplitude bias), and one measure of saccadic speed (latency).

#### Direction bias

**Fig. 2:**
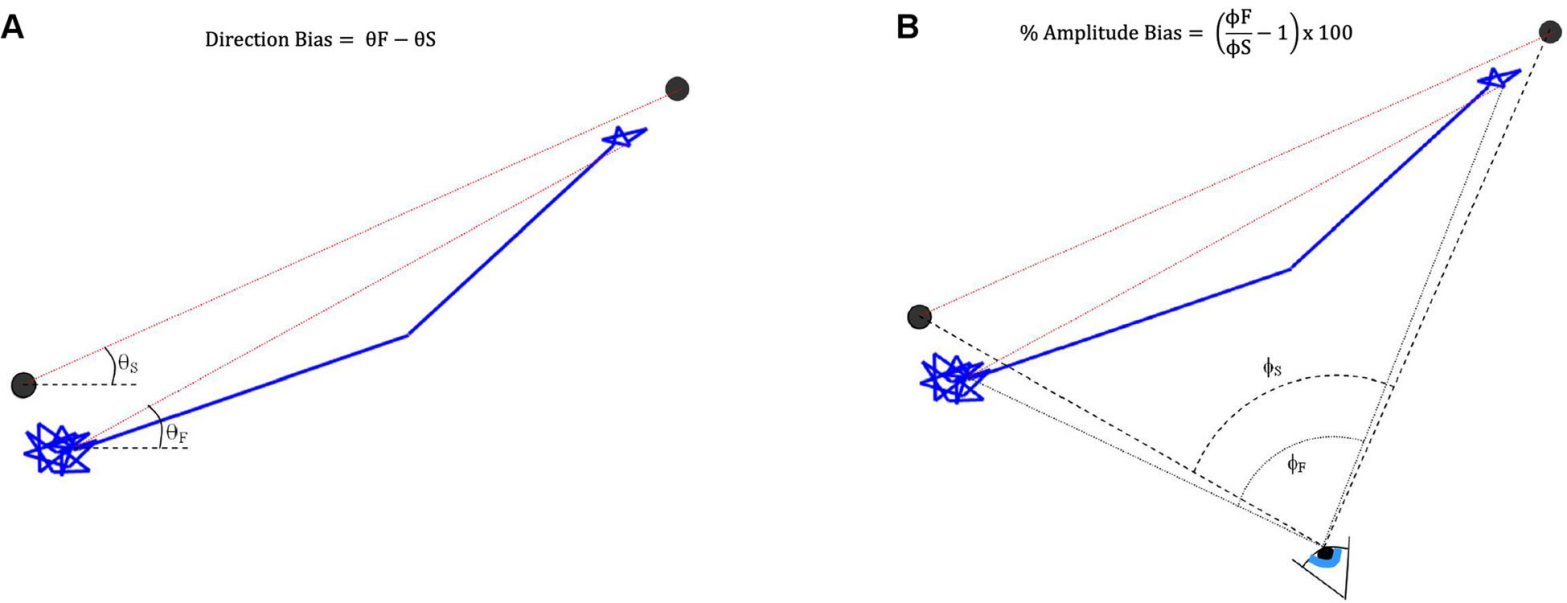
Examples of calculation of (A) direction bias (in degrees) and (B) amplitude bias (in percent)

Direction bias (in degrees) was defined as the difference between the fixation change direction and the stimulus change direction (Fig. 2A). The fixation change direction was calculated as the angle between a horizontal line extending from the fixation change start point, and the line of the fixation change in an anti-clockwise direction, whereas the stimulus change direction was calculated as the angular direction between a line between the centre of the fixation stimulus point and the centre of the test stimulus point (stimulus change direction) and the same horizontal reference line. A positive direction bias therefore represented a fixation change direction greater than the stimulus change direction.

#### Amplitude bias

The amplitude of detected fixation change was calculated as a visual angle subtended at the eye. The visual angle corresponding to the fixation change was calculated by using the eye positional data (x, y and z coordinates) at the start of the detected fixation change, which could be calculated from the distance data provided by the eye tracker corresponding to the start point data sample. As the eye position is known along with the position of the start and end points of the fixation change, the angle of the fixation change (at the eye) could be calculated (Fig. 2B). Amplitude bias was defined as the bias between the fixation change angle and the test stimulus angle in percent difference. Positive values represented a fixation change angle which was larger than the test stimulus visual field angle, and negative values represented a fixation change angle which was less than the displayed test stimulus visual field angle.

#### Latency

Latency was calculated (in milliseconds) simply by taking the time difference between the time the test stimulus was initially presented on the display screen and the time at the beginning of the first detected fixation change subsequent to a test stimulus being displayed.

### Statistical analysis

The distribution of results was examined using histograms and Shapiro-Wilk test. Student-t test was used for comparison of normally distributed variables, with Wilcoxon rank sum test used for non-parametric variables. Scatter plots were used to examine the relationship between average SVOP sensitivity and eye movement parameters including saccade latency, standard deviation of saccade latency, amplitude bias and direction bias. Univariable and multivariable regression analysis was then performed to examine the relationship between eye movement parameters from SVOP and average SVOP sensitivity, average SAP sensitivity, and age. Logistic regression was also used to examine the odds of glaucoma associated with SVOP sensitivity, amplitude bias, direction bias, average latency, latency standard deviation and age.

Receiver Operating Characteristic (ROC) curves were constructed to assess the ability of SVOP eye movement parameters to differentiate participants with glaucoma from healthy controls, with results compared to SVOP and SAP mean sensitivity. The area under the ROC curve (AUC), adjusted for age differences between cases and controls, was used to summarize the diagnostic accuracy [19]. ROC regression used a 1000 repetition bootstrap technique to estimate 95% confidence intervals. Results for all left eyes were transposed to right eye formats and compared between eyes with and without glaucoma. All statistical analyses were performed with commercially available software (Stata version 14; StataCorp LP, College Station, TX). The α level (type I error) was set at 0.05.

## Results

**Fig. 3:**
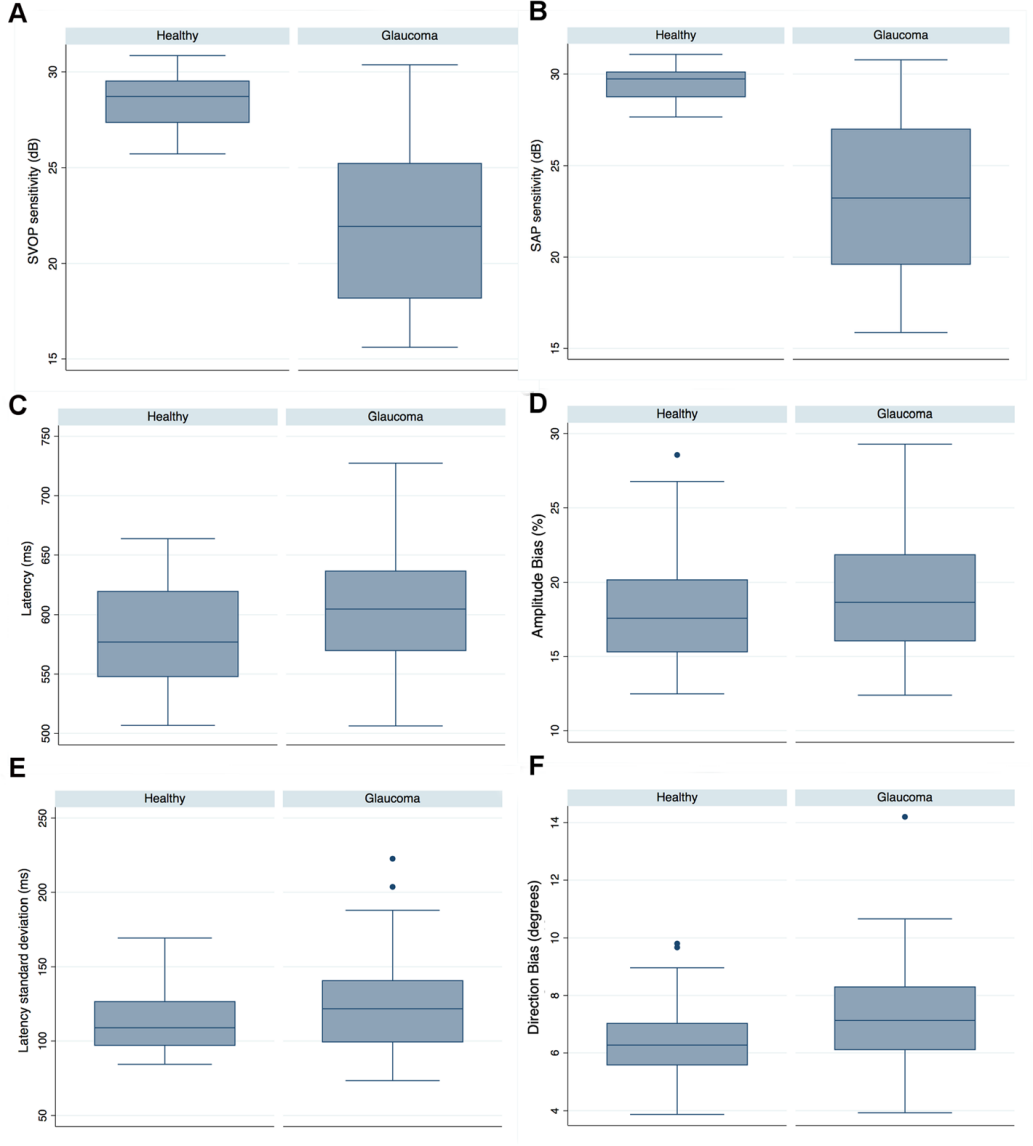
Box plots comparing threshold sensitivity and eye movement parameters (latency, latency standard deviation, amplitude bias and direction bias) obtained from saccadic vector optokinetic perimetry (SVOP) and standard automated perimetry (SAP) in healthy and glaucomatous eyes

After exclusion of unreliable results, 46 eyes of 23 healthy subjects and 61 eyes of 31 patients with glaucoma were included in the analysis. Demographic and clinical characteristics of participants are summarised in Table 1. Participants with glaucoma were slightly older than controls (72.3 ± 7.9 compared to 65.9 ± 5.6 years, *P* < 0.001). Twenty eight participants (51.9%) were female. Eyes with glaucoma had a mean ± SD SAP mean deviation (MD) of − 8.72 ± 7.37 dB, with average SVOP and SAP sensitivities of 22.11 ± 4.25 and 23.28 ± 4.39 dB respectively. SVOP and SAP sensitivities were significantly lower in eyes with glaucoma compared to controls (Table 1, Fig. 3A and B). Patients with glaucoma also had significantly longer latency of saccades, with a mean of 602.9 ± 50.0 ms in those with glaucoma compared to 578.3 ± 44.6 ms for controls (*P* = 0.009) (Fig. 3C). Eyes with glaucoma also had reduced accuracy of saccades, with an average direction bias of 7.4 ± 1.8 degrees compared to 6.5 ± 1.5 degrees in healthy subjects (*P* = 0.006) (Fig. 3F). There was no difference in the standard deviation of latency of saccades or in amplitude bias (Table 1, Fig. 3D and E).

**Table 1:** Demographic and clinical characteristics of study participants

	Healthy 46 eyes	Glaucoma 61 eyes	***P***-value
**Age (years)**	65.9 ± 5.6	72.3 ± 7.9	< 0.001
**Gender**	14 female, 9 male	14 female, 17 male	0.171
**SAP MD (dB)**	−0.02 ± 0.84	−8.72 ± 7.37	< 0.001
**Average SAP sensitivity (dB)**	29.55 ± 0.90	23.28 ± 4.39	< 0.001
**Average SVOP sensitivity (dB)**	28.40 ± 1.29	22.11 ± 4.25	< 0.001
**Average latency (ms)**	578.3 ± 44.6	602.9 ± 50.0	0.009
**Latency standard deviation (ms)**	114.0 ± 22.3	123.5 ± 31.2	0.084
**Amplitude bias (%)**	17.9 ± 3.8	19.0 ± 4.0	0.173
**Direction bias (degrees)**	6.5 ± 1.5	7.4 ± 1.8	0.006

**Fig. 4:**
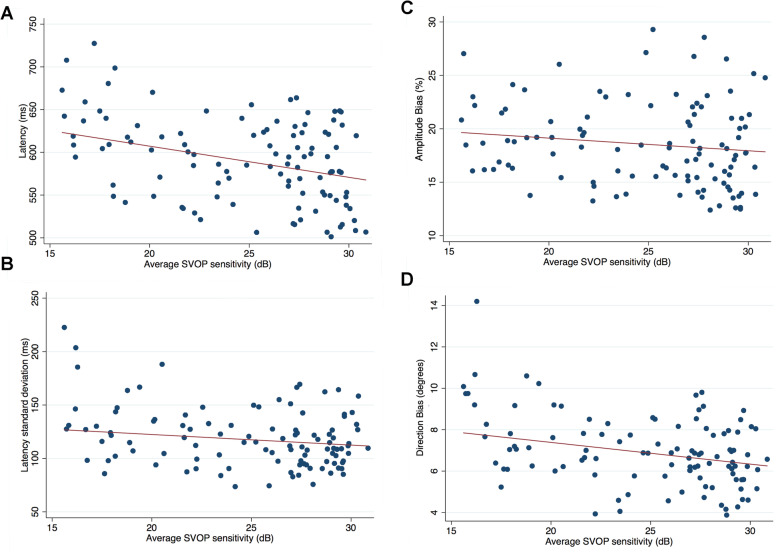
Scatter plots showing the relationship between average saccadic vector optokinetic perimetry (SVOP) sensitivity and latency of saccades (A), latency standard deviation (B), amplitude bias (C) and direction bias (D)

There was a significant increase in saccade latency with worsening SVOP and SAP sensitivity (Table 2, Fig. 4A). Each 1 dB decrease in SVOP sensitivity was associated with a 4.23 ms (95% CI 2.31 to 6.14) increase in latency (*P* < 0.001) and each 1 dB decrease in SAP sensitivity was associated with a 3.67 ms (95% CI 1.73 to 5.62) increase in latency. Latency also increased with age, with a 1.34 ms (95%CI 0.13 to 2.55) increase per year older (*P* = 0.030). Accounting for age in a multivariable model, the relationship between worsening SVOP and SAP sensitivity and longer latency of saccades remained (Table 3).

**Table 2:** Univariable analysis examining the relationship between average SVOP sensitivity, age and average SAP sensitivity and eye movement parameters obtained from SVOP

	Coefficient	95% CI	P	R^**2**^	P
**Average SVOP sensitivity**					
**Amplitude Bias (%)**	−0.15	−0.32 to 0.01	0.066	0.032	0.066
**Direction Bias (degrees)**	−0.16	−0.22 to − 0.09	< 0.001	0.171	< 0.001
**Latency (ms)**	−4.23	−6.14 to −2.31	< 0.001	0.154	< 0.001
**Latency standard deviation (ms)**	−1.86	−2.99 to −0.73	0.002	0.092	0.002
**Age**					
**Amplitude Bias (%)**	0.08	−0.02	0.123	0.023	0.123
**Direction Bias (degrees)**	0.05	0.01 to 0.09	0.027	0.046	0.027
**Latency (ms)**	1.34	0.13 to 2.55	0.030	0.044	0.030
**Latency standard deviation (ms)**	0.66	−0.03 to 1.36	0.061	0.033	0.061
**Average SAP sensitivity**					
**Amplitude Bias (%)**	−0.15	− 0.31 to 0.01	0.073	0.030	0.073
**Direction Bias (degrees)**	−0.16	−0.23 to − 0.09	< 0.001	0.180	< 0.001
**Latency (ms)**	−3.67	−5.62 to −1.73	< 0.001	0.118	< 0.001
**Latency standard deviation (ms)**	−1.92	−3.04 to −0.79	0.001	0.098	0.001

**Table 3:** Multivariable analysis examining the relationship between average SVOP sensitivity and average SAP sensitivity and eye movement parameters obtained from SVOP, accounting for age

Average SVOP sensitivity			
	Coefficient	95% CI	P
**Direction Bias (degrees)**	−0.16	−0.08 to − 0.25	< 0.001
**Age**	− 0.001	− 0.06 to 0.04	0.743
**Latency (ms)**	−4.38	−6.73 to −2.02	< 0.001
**Age**	−0.15	−1.55 to 1.25	0.829
**Latency standard deviation (ms)**	−1.82	−0.43 to −3.21	0.011
**Age**	0.04	−0.79 to 0.87	0.922
Average SAP sensitivity			
	**Coefficient**	**95% CI**	**P**
**Direction Bias (degrees)**	−0.17	−0.09 to − 0.25	< 0.001
**Age**	−0.01	− 0.06 to 0.04	0.739
**Latency (ms)**	−3.52	−5.89 to −1.15	0.004
**Age**	0.16	−1.25 to 1.58	0.820
**Latency standard deviation (ms)**	−1.89	−3.25 to −0.52	0.007
**Age**	0.03	−0.78 to 0.85	0.939

There was also a significant increase in direction bias with worsening SVOP and SAP sensitivity (Table 2, Fig. 4D). Direction bias increased by 0.16 degrees (95% CI 0.09 to 0.22 degrees) for each 1 dB worse SVOP sensitivity (*P* < 0.001) and by 0.16 degrees (95% CI 0.09 to 0.23 ^o^) for each 1 dB worse SAP sensitivity (P < 0.001). The relationship remained after accounting for age (Table 3). There was also greater variation in latency of saccades with worsening SVOP (Fig. 4B) and SAP sensitivity, with the standard deviation of latency measurements increasing by 1.86 ms (95% CI 0.73 to 2.99) for each 1 dB worse SVOP sensitivity (*P* = 0.002) and by 1.92 ms (95% CI 0.79 to 3.04) for each 1 dB worse SAP sensitivity (*P* = 0.001). These relationships also remained after accounting for age (Table 3).

In univariable analysis, factors associated with increased odds of glaucoma included worse SVOP sensitivity, longer latency of saccades, and older age (Table 4). However, in the multivariable model only SVOP sensitivity was significant, suggesting that eye movement latency data did not provide additional value compared to SVOP sensitivity alone for differentiating eyes with glaucoma from healthy controls (Table 4).

**Table 4:** Univariable and multivariable logistic regression analysis showing the odds of glaucoma for average SVOP sensitivity, amplitude bias, direction bias, average latency, latency standard deviation and age

	Univariable Analysis			Multivariable Analysis		
	Coefficient	95% CI	P-value	Coefficient	95% CI	P-value
**SVOP sensitivity (dB)**	2.076	1.534 to 2.810	< 0.001	2.102	1.492 to 2.961	< 0.001
**Amplitude Bias (%)**	1.013	0.986 to 1.040	0.346	NA	NA	NA
**Direction Bias (degrees)**	1.021	0.9745 to 1.070	0.378	NA	NA	NA
**Average Latency (ms)**	1.011	1.002 to 1.020	0.012	1.004	0.989 to 1.018	0.796
**Latency standard deviation (ms)**	1.013	0.998 to 1.028	0.088	1.004	0.979 to 1.030	0.762
**Age (years)**	1.138	1.069 to 1.212	< 0.001	0.990	0.893 to 1.090	0.796

**Fig. 5:**
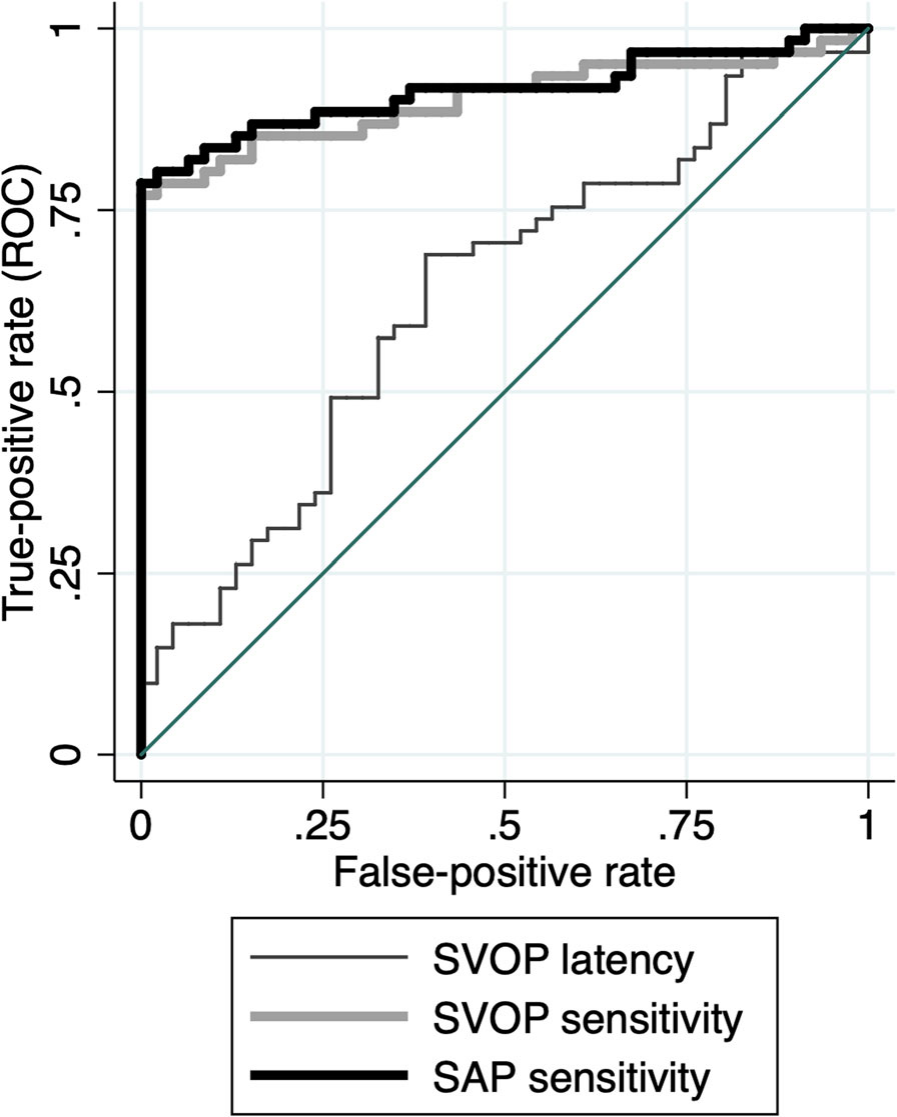
Receiver operating characteristic curves showing the ability of latency of saccades, saccadic vector optokinetic perimetry (SVOP) sensitivity and SAP sensitivity to differentiate eyes with glaucoma from healthy controls

**Fig. 6:**
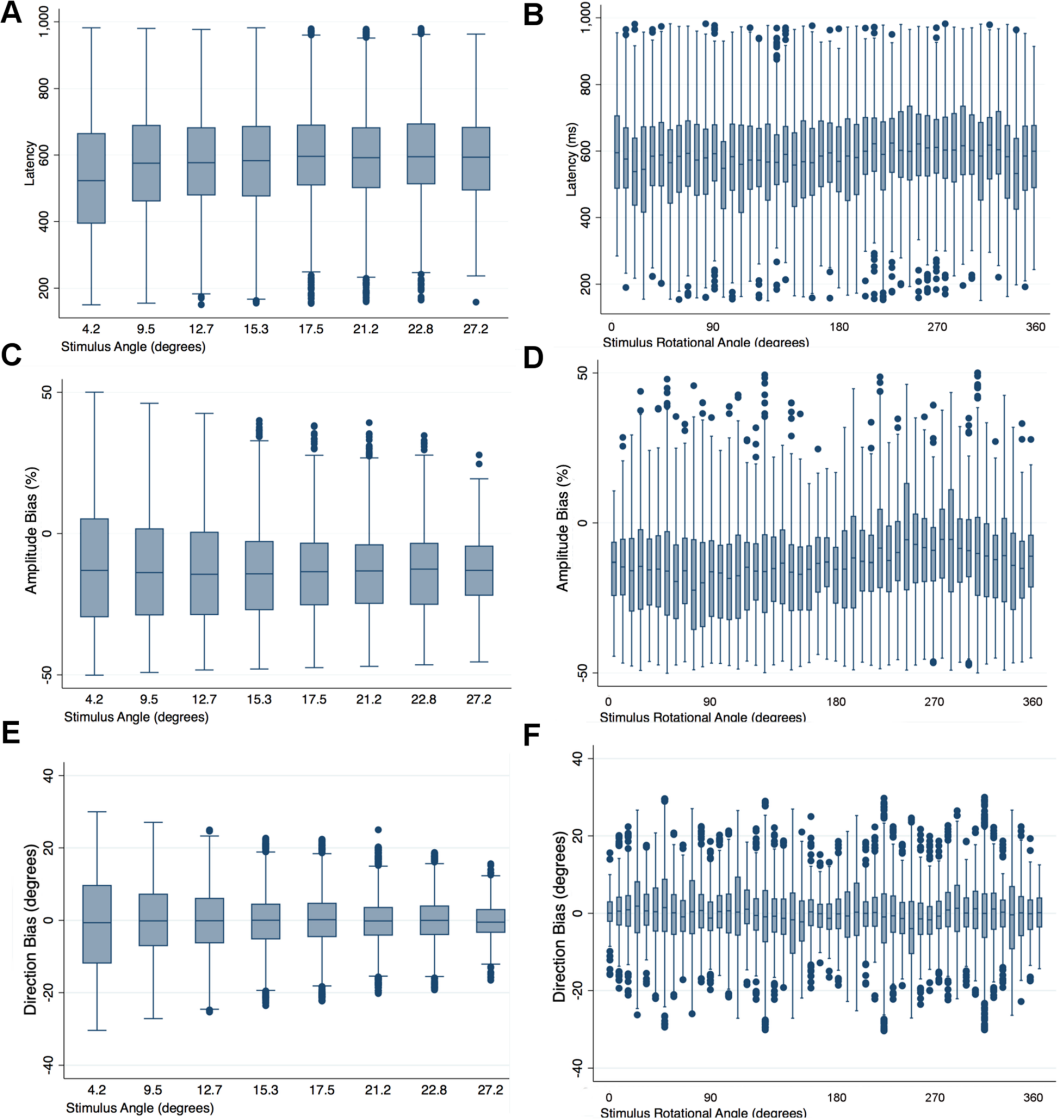
Box plots showing the point wise relationship between latency of saccades and stimulus angle (**a**) and stimulus rotational angle (**b**); between amplitude bias and stimulus angle (**c**) and stimulus rotational angle (**d**); and between direction bias and stimulus angle (**e**) and stimulus rotational angle (**f**)

SAP and SVOP sensitivity had excellent ability to differentiate glaucomatous and healthy eyes, with AUCs of 0.914 (95%CI 0.856 to 0.973) and 0.902 (95% CI 0.838 to 0.966) (Fig. 5). In contrast, latency of saccades had an AUC of only 0.635 (95%CI 0.527 to 0.742). There was no significant difference in AUCs for SVOP and SAP sensitivity (*P* = 0.564). Average latency was significantly worse at differentiating glaucomatous and healthy eyes than SAP sensitivity (*P* < 0.001) and SVOP sensitivity (P < 0.001). The AUC for latency deviation was 0.597 (95% CI 0.485 to 0.709), which was also significantly worse than SAP and SVOP sensitivities (P < 0.001 for both comparisons). Pointwise analysis revealed no significant relationship between latency of saccades and stimulus angle or stimulus rotational angle (Fig. 6A and B), or between amplitude bias and stimulus angle or stimulus rotational angle (Fig. 6C and D), or direction bias and stimulus angle or stimulus rotational angle (Fig. 6E and F).

## Discussion

The results of this study demonstrate a significant difference in eye movement patterns in patients with glaucoma compared to controls, with patients with glaucoma having slower and less accurate saccades, evident by increased direction bias. There was no significant difference in amplitude bias or the standard deviation of latency of saccades; however, there was a relationship between higher standard deviation of latency and worse SAP and SVOP sensitivity (Table 2). Though patients with glaucoma were on average older than controls, the association between worse visual field sensitivity and longer saccade latency, higher direction bias, and increased standard deviation of latency, remained after accounting for age in a multivariable model (Table 3).

Previous studies have also demonstrated altered eye movements in patients with glaucoma, suggesting that measurement of eye movements might be useful for detecting glaucoma or assessing the impact of glaucoma on ability to perform activities of daily living or quality of life [7] [20]. [21, 22] Latency of saccades towards stimuli at eccentricities and distances corresponding to the 54 test locations of the 24–2 HFA test pattern have also been previously examined in patients with glaucoma [23]. Mazumdar et al. examined 25 patients with glaucoma using an eye movement perimeter and reported delayed initiation of saccadic eye movements in patients with glaucoma and a trend towards longer latency with worse glaucoma severity, however results were only reported for testing with a single contrast stimulus and no other eye movement parameters were assessed. To the best of our knowledge no studies have combined an analysis of eye movement patterns with simultaneous assessment of threshold visual field sensitivities. The ability of SVOP to provide information on eye movement patterns at the same time as determining differential light sensitivity offers the opportunity to simultaneously assess two aspects of visual function known to be affected by glaucoma.

Accounting for age, there was a 4.38 ms (95% CI 2.02 to 6.73) increase in latency of saccades; a 0.16 degree (95% CI 0.08 to 0.25 degrees) increase in direction bias; and a 1.82 ms (95% CI 0.43 to 3.21) increase in latency standard deviation for each 1 dB worsening SVOP sensitivity. However, as SVOP determines whether or not a stimulus has been seen by eye tracking, a relationship between eye movement parameters and SVOP sensitivity might be expected. It was therefore important to also examine the relationship between eye movements and severity of glaucoma measured from SAP. A similar relationship was observed with, accounting for age, a 3.52 ms (95% CI 1.15 to 5.89) increase in latency of saccades; a 0.17 degree (95% CI 0.09 to 0.25 degrees) increase in direction bias; and a 1.89 ms (95% CI 0.52 to 3.25) increase in latency standard deviation for each 1 dB worsening SAP sensitivity (Table 3).

Latency was measured as the time difference between the time of stimulus presentation to the beginning of the first detected fixation change subsequent to a test stimulus being displayed. As during SVOP testing, the fixation spot is the preceding stimulus, the distance between the point of fixation and stimulus varies during testing and varies from test to test. The relationship between latency and the spatial location of stimuli presented during testing was examined but there was no significant association (Fig. 6a and b). There was also no significant effect of stimulus location on amplitude bias, though for direction bias, the spread of values diminished with stimuli presented at a greater angle (Fig. 6e).

Previous studies have also shown higher latency of saccades in patients with glaucoma. For example, Lee and colleagues found older drivers with glaucoma had delayed fixation times compared to similarly aged controls when trying to identify hazards during simulated driving [21]. The outcome of delayed fixation time is similar to our finding of reduced latency. Others have reported changes such as reduced fixation rates [7], reduced saccade rate [12] and longer fixation durations [7], illustrating the large number of eye movement parameters potentially affected by glaucoma.

It has been proposed that information from assessment of eye movements might be useful for detecting glaucoma [7]. We therefore examined the relationship between eye movement measures and odds of glaucoma and examined AUCs using ROC analysis. Increased latency of saccades was associated with increased odds of glaucoma; however, eye movement measures were no longer significant when accounting for threshold sensitivity values from SVOP (Table 4). In addition, latency of saccades had an AUC of only 0.635, compared to 0.914 for SVOP sensitivity. These results suggest that inclusion of information regarding latency of saccades from SVOP is likely to provide no additional value for glaucoma detection compared to SVOP sensitivity values alone. However, further study is warranted to examine whether some patients may exhibit changes to eye movements prior to changes in differential light sensitivity [13] and to examine the relationship between eye movements and ability to perform tasks of daily living and quality of life. SVOP may provide a useful tool to obtain eye movement data for further study.

Limitations of the present study include that healthy participants were significantly younger than those with glaucoma; however, age was accounted for in the multivariable analyses. In addition, patients were not categorised based on the location of visual field loss, and so it was not possible to determine variation in eye movements relative to areas of damage or preserved visual field. We also only examined 4 eye movement-related parameters as these are the parameters automatically generated by the SVOP experimental device. It is also important to acknowledge that SVOP does not attempt to simulate an activity of daily living and therefore studies which examine eye movements during realistic tasks may provide greater information about the impact of altered eye movements on quality of life and therefore be of greater relevance to patients. In future studies it would be interesting to examine the relationship between eye movement metrics derived from SVOP and those during real world tasks such as reading or driving.

## Conclusions

In conclusion, this study identified significant differences in eye movements between patients with glaucoma and healthy individuals. Those with glaucoma had longer latency and less accurate saccades and there was a relationship between altered saccades and worse glaucoma severity. SVOP enabled information regarding eye movements to be determined at the same time as assessment of visual field threshold sensitivity values, however, eye movement parameters were not of additional benefit in differentiating eyes with glaucoma from healthy controls.

## Data Availability

The datasets generated during and/or analysed during the current study are available from the corresponding author on reasonable request.

## Abbreviations

AUC: Area under the receiver operating characteristic curve
dB: Decibels
IOP: Intraocular pressure
LCD: Liquid Crystal Display
MD: Mean deviation
SAP: Standard automated perimetry
SVOP: Saccadic vector optokinetic perimetry
